# Researchers, patients, and other stakeholders’ perspectives on challenges to and strategies for engagement

**DOI:** 10.1186/s40900-020-00227-0

**Published:** 2020-10-07

**Authors:** Andrea Heckert, Laura P. Forsythe, Kristin L. Carman, Lori Frank, Rachel Hemphill, Emily A. Elstad, Laura Esmail, Julie Kennedy Lesch

**Affiliations:** 1 Present Address, Independent Consultant, Portland, OR USA; 2 Evaluation & Analysis, Patient-Centered Outcomes Research Institute, 1828 L St NW, Suite 900, Washington, DC 20036 USA; 3 Public and Patient Engagement, Patient-Centered Outcomes Research Institute, Washington, DC 20036 USA; 4 RAND, Arlington, VA 22202 USA; 5 American Institutes for Research, Chapel Hill, NC 27517 USA; 6 Present Address, Independent Consultant, Paris, France; 7 Clinical Effectiveness and Decision Science, Patient-Centered Outcomes Research Institute, 1828 L St NW, Suite 900, Washington, DC, USA

**Keywords:** Engagement challenges, Engagement strategies, Comparative effectiveness research, Patient-centered research, Stakeholder engagement, Stakeholder participation, Patient engagement, Patient participation

## Abstract

**Background:**

There is growing interest in patient and stakeholder engagement in research, yet limited evidence about effective methods. Since 2012, the Patient-Centered Outcomes Research Institute (PCORI) has funded patient-centered comparative effectiveness research with a requirement for engaging patients and other stakeholders as research partners in study planning, conduct, and dissemination. This requirement, unique among large healthcare research funders in the US, provides an opportunity to learn about challenges encountered and specific strategies used by PCORI-funded study teams. The primary objective of this study is to describe -- from the perspective of PCORI investigators and research partners—the most common engagement challenges encountered in the first two years of the projects and promising strategies to prevent and overcome these challenges.

**Methods:**

Descriptive information about investigators, partners, and their engagement was collected from investigators via annual (*N* = 235) and mid-year (*N* = 40) project progress reporting to PCORI, and from their partners (*N* = 260) via voluntary survey. Qualitative data were analyzed using content and thematic analyses.

**Results:**

Investigators and partners most often described engagement challenges in three domains: (1) infrastructure to support engagement, (2) building relationships, and (3) maintaining relationships. Infrastructure challenges related to financial and human resources, including funding support and dedicated staff, identifying diverse groups of partners, and partners’ logistical needs. Challenges for both building and maintaining relationships encompass a variety of aspects of authentic, positive interactions that facilitate mutual understanding, full participation, and genuine influence on the projects. Strategies to prevent or mitigate engagement challenges also corresponded overall to the same three domains. Both groups typically described strategies more generally, with applicability to a range of challenges rather than specific actions to address only particular challenges.

**Conclusion:**

Meaningful engagement of patients and other stakeholders comes with challenges, as does any innovation in the research process. The challenges and promising practices identified by these investigators and partners, related to engagement infrastructure and the building and maintenance of relationships, reveal actionable areas to improve engagement, including organizational policies and resources, training, new engagement models, and supporting engagement by viewing it as an investment in research uptake and impact.

## Plain English summary

There is growing interest in research that involves patients and other stakeholders as partners in study planning, conduct, and sharing information about health research results (known in the US as “engagement”). Yet, we know little about which engagement methods work best. Understanding the challenges that stakeholders and researchers experience when working together can help uncover promising practices. Since 2012, the Patient-Centered Outcomes Research Institute (PCORI) has funded patient-centered research that compares the benefits and harms of alternative methods for prevention, diagnosis, treatment, or delivery of care. PCORI has required that investigators engage with patients and other stakeholders in the research process. We reviewed 235 yearly project reports and 40 mid-year reports from researchers and 260 voluntary surveys from stakeholder partners. We aimed to describe common engagement challenges that the teams experienced in the first two years of their projects and promising strategies used to respond to these challenges. We found three areas of engagement challenges [1]: infrastructure to support engagement [2], building relationships, and [3] maintaining relationships. Infrastructure challenges included ensuring financial support and staff time, finding a diverse group of patient and stakeholder partners, and recognizing partner needs. Challenges in building and maintaining relationships included how investigators and stakeholders communicate and share ideas when working together on research projects. Researchers and stakeholders described strategies in the same three areas. Results help identify ways that more resources, training, and policies, as well as researcher and stakeholder practices, make engagement in research easier and more effective.

## Background

Involving patients and the public as partners in health research is a key strategy to produce more useful, patient-centered evidence to guide healthcare decisions. In the UK, involvement of patients and members of the public in prioritizing, planning, and conducting research is commonly called ‘patient and public involvement’ (PPI) and involvement in dissemination and knowledge sharing is called ‘engagement’ [1]. Meanwhile in the US, particularly for studies funded by the Patient-Centered Outcomes Research Institute (PCORI) (the context for this study), the term ‘patient and stakeholder engagement in research’ encompasses involvement of patients and other health care stakeholders along the entire spectrum of research activities up to and including dissemination. Further, engagement also includes other health care stakeholders (such as clinicians, policymakers, representatives of healthcare systems, and organizations that purchase or provide insurance) [2, 3] to better align pragmatic clinical research with both patients’ and other stakeholders’ real-world needs and concerns [4]. Despite differences in terms and the range of communities involved, there are many commonalities for the rationale and approaches for involving patients and others partners in research around the globe. For example, approaches to partner involvement in research fall on a continuum, from one-way input to two-way collaboration to shared study leadership or co-production [5–7].

Growing evidence indicates that engagement in healthcare research contributes to increased relevance of study topics and outcomes for patients and other stakeholders; better study participant enrollment and retention; and improved sharing of findings [8–15]. However, despite the burgeoning literature on engagement, little evidence exists about the specific methods to effectively engage patients and other stakeholders as meaningful partners in research [16, 17]. Reports about challenges, promising engagement practices, and lessons learned typically focus on an individual study team’s experience (e.g. [18–24],) or small groups of studies, often on specific topics or geographic areas [25–28]. Review articles about engagement in research typically summarize some broad challenges [13, 15, 29–33] and focus on describing engagement characteristics and the impact of this engagement. Often, the depth and breadth of real-world barriers and facilitators are not explored.

Further, perhaps the most important limitation of most existing information about engagement experiences is that it largely comes from researchers alone rather than patient and other stakeholder partners. To more effectively address the engagement challenges that matter most to partners specifically and to improve their experiences, we need to learn directly from them what they view as most challenging and helpful for engagement. Ultimately, richer information on engagement from larger samples that include patient and other stakeholder partners will allow researchers, engaged partners, funders, and policymakers to better understand, mitigate, and prevent challenges to stakeholder engagement in research.

The growing body of research projects funded by PCORI, along with PCORI’s funding requirements and broad engagement guidance [34], create a shared context to study engaged research on a large scale. The US Congress established PCORI in 2010 with a mandate to provide patients and other stakeholders with useful evidence to make more informed health decisions. PCORI funds comparative clinical effectiveness research (CER) that compares the benefits and harms of interventions for prevention, diagnosis, treatment, or care delivery. PCORI-funded studies examine interventions focused on patients, caregivers, healthcare providers, and/or health systems policies, and are intended to be pragmatic and applicable to real-world settings. As such, PCORI also requires engagement of patients and other stakeholders as research partners in all funded CER—a unique practice among large US clinical research funders. PCORI provides broad guidance about how to engage partners [34]. This approach allows research teams to explore engagement practices and address challenges on their own terms, with their available resources, and specific to their institutional and project context. Collectively, these projects serve as a laboratory of innovation that generated practice-based evidence about engagement across a wide range of projects and approaches to engagement.

This study describes the most common engagement challenges encountered in the first two years of the projects and promising strategies to prevent and overcome these challenges. This study is distinguished from prior engagement literature by capturing the perspectives of relatively large samples of both patients and other stakeholder research partners as well as investigators. These perspectives were elicited from investigators and partners from PCORI’s earliest funded research projects who had a wide range of backgrounds and levels of experience with engaged research and implemented various engagement approaches.

## Methods

The MaGil (now Advarra) Institutional Review Board (IRB) completed the ethical review and approved this project.

### Data sources

This study used data from investigators and patient and other stakeholder research partners. No existing validated instruments were available to measure engagement challenges and strategies for either sample. The engagement questions were informed by past efforts [10], PCORI’s conceptual model of patient-centered outcomes research [4], the PCORI Evaluation Framework 2.0 [35], peer-reviewed literature, and guidance from PCORI stakeholders (described below).

The main source of investigator data was non-anonymous progress reports required for all PCORI research projects. As part of an annual progress report [36] completed at the end of each project year, investigators responded to a wide set of closed- and open-ended questions including an open-ended question eliciting information about any engagement challenges and current and future strategies to overcome them. They also completed closed-ended descriptive questions about engagement in their PCORI project that provide context for interpreting the current analyses, including community types engaged (e.g., patients, clinicians), phases of the project in which partners were engaged (e.g., research topics or questions, study participant recruitment), and the engagement approaches implemented (e.g., advisory group, research team member). Investigators completed similar questions on a shorter progress report collected every 6 months (see supplemental Appendix A for both annual and mid-year questions analyzed for this study). Additional self-reported demographic information about investigators, including their sex and experience with research, was retrieved from research applications.

Partner-provided data, unlike investigator data, were both confidential and voluntary and thus they represent a subset of funded PCORI projects. Partners were identified for this study by asking investigators, after they completed the annual progress report, to nominate up to 10 partners to share their experiences with engagement. Nominated partners were invited to respond to the Ways of Engaging-ENgagement ACtivity Tool (WE-ENACT) [37] via web survey or, if preferred, by telephone interview. The tool includes open-ended questions about engagement challenges similar to those completed by the investigator, as well as facilitators to and suggestions for meaningful engagement (see supplemental Appendix A for questions analyzed for this study). To describe the study sample, the tool also includes closed-ended items about the phases of the project in which the partner was engaged, the community they primarily represent, and other demographics (age, sex, race, ethnicity, and educational attainment). A detailed qualitative analysis of both investigator and partner responses to other open-ended questions about partner contributions to study planning and conduct and the influence of these contributions to the PCORI projects is published elsewhere [11].

### Data collection and sampling

**Fig. 1:**
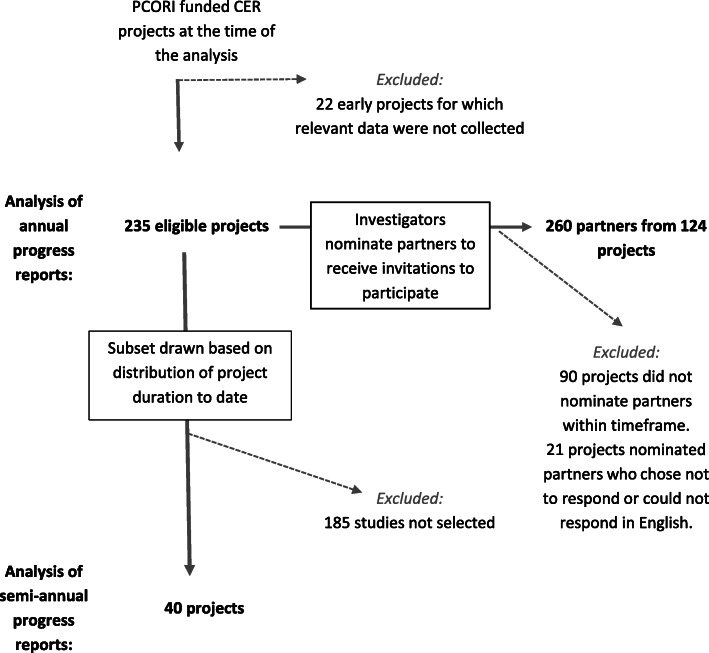
Investigator and partner samples

Designed to look at early PCORI investigator experiences with engagement, the investigator sample includes all eligible projects that submitted annual progress reports between July 1, 2015 and June 30, 2016 for either the first project year (year 1) or the second (year 2). Because PCORI was a new and maturing organization, at the time of the analysis, most of the projects were within their first two years, while a small number of projects (*n* = 22) were excluded because they were already in their third (and final) project year. This sample represents 91% of relevant projects funded by PCORI at this time. Additionally, a smaller purposive subsample of 40 projects was selected to examine additional responses about engagement challenges and strategies to overcome them from the mid-year progress reports. The subsample was selected to include equal numbers of projects at different stages of progress covering a minimum of the first 12 months and a maximum of the first 30 months of the project (i.e., that submitted two, three, four, or five consecutive mid-year reports). The partner sample included all available year 1 and year 2 WE-ENACT surveys collected between July 1, 2015 and June 30, 2016. Multiple aspects of our approach and sampling design (e.g., not a one-to-one match between investigators and partners, investigators responding about engagement in the project collectively with partners responding about their individual experiences) preclude a matched comparison of investigator and partner perspectives from the same projects (see Fig. 1).

### Analysis

Basic descriptive information about investigators, partners, and their engagement was calculated using frequencies and means. For qualitative analyses, we first examined open-ended responses from the investigators’ annual progress reports and partners’ WE-ENACT surveys concurrently via content and thematic analysis [38, 39]. We applied deductive and inductive codes to relevant text using NVivo v11 [40]. To ensure inter-coder agreement, three analysts independently coded 10% of the same data and reconciled discrepancies. Coded text occurrence queries were generated to establish theme prominence using a 10% minimum threshold. Themes among the partner data were further examined for differences by community stakeholder type (patients/consumers, caregivers, and advocacy or community organizations vs. all other stakeholders). We then sought to enrich the findings from these first two analyses and synthesize the challenges and strategies [41]. We analyzed investigators’ responses to the mid-year report questions - specifically about engagement challenges and strategies to overcome them - from the purposive subsample of 40 projects in order to inductively identify additional engagement challenges and strategies and to explore patterns within and across reporting periods. Analysts recorded relationships between these investigator descriptions and the earlier analyses.

### Stakeholder engagement

PCORI’s Advisory Panel on Patient Engagement (PEAP) [42] helped conceptualize and design this study with the aim of ensuring the study questions reflected stakeholder priorities and that the results would provide actionable information. The PEAP is comprised of patients, caregivers, clinicians, and organizations representing these stakeholders, who are diverse in race, ethnicity, gender, sexual identity, immigration status, geography, and other characteristics [43]. These advisors contributed to this work in multiple ways, including participating in the development of the evaluation framework and conceptual model that guided the work, highlighting the importance of the specific research aims, ensuring that PCORI collected important engagement insights from patient and other stakeholder partners, recommending specific survey questions to include, and contributing to the final interpretation of findings.

## Results

### Sample description

#### Investigator sample

The investigator sample included investigator reports from 235 PCORI research projects: 91 reports from year 1 and 144 reports from year 2 (Table 1). Although most investigators (96%) were reporting on their first PCORI award, the sample of investigators were experienced researchers. Three-quarters of investigators reported 10 or more years of research experience at the time of application, and 42% had previously been principal investigators on more than 10 research studies awarded by other funders. Most investigators reported working with research partners representing the patient/consumer (88%) and clinician (89%) perspective (Table 2). Investigators reported engaging with an average of 4.9 (±2.0) different stakeholder partner types (range 1 to 11). Investigators most commonly reported engaging with research partners through advisory groups (82%) and as research team members (81%), with more than half (56%) reporting that research partners served as co-investigators. Investigators reported engaging with partners across all study phases, from refining research topics to selecting outcomes to sharing study findings.

**Table 1:** Investigator-reported characteristics (*N* = 235 investigators)

Characteristic	n (%)
**Sex** (n, %)	
Female	112 (48%)
Male	123 (52%)
**Research experience** ^a^ (n, %)	
0–4 years	12 (5%)
5–9 years	45 (19%)
10+ years	176 (76%)
**Previous funded studies as principal investigator** ^b^ (n, %)	
0	4 (2%)
1–5	81 (35%)
6–10	52 (22%)
11–15	30 (13%)
16–20	21 (9%)
21+	46 (20%)

^a^Item completed at time of application submission: How many years of research experience do you have related to this field of research? Response is missing for 2 investigators

^b^Item completed at time of application submission: Approximately how many grants/contracts have you had funded as the PI or project lead? Response is missing for 1 investigator

**Table 2:** Investigator-reported description of partner engagement in their projects (N = 235 projects)

Characteristics	Year 1 progress reports (*n* = 91)	Year 2 progress reports (*n* = 144)	Total (N = 235)
**Partner communities engaged** ^**a**^(n, %)			
Clinician	83 (91%)	126 (88%)	209 (89%)
Patient/consumer	82 (90%)	125 (87%)	207 (88%)
Patient/caregiver advocacy organization	56 (62%)	84 (58%)	140 (60%)
Clinic/hospital/ health system representative	53 (58%)	81 (56%)	134 (57%)
Caregiver/family member of patient	43 (47%)	77 (53%)	120 (51%)
Subject matter expert^b^	43 (47%)	78 (54%)	121 (51%)
Training institution representative^c^ (non-research health professions including educator)	15 (16%)	22 (15%)	37 (16%)
Policy maker (government official)	10 (11%)	28 (19%)	38 (16%)
Payer (public or private insurance)	13 (14%)	22 (15%)	35 (15%)
Life sciences industry representative	2 (2%)	9 (6%)	11 (5%)
Purchaser of insurance plans (small or large employers)	0 (0%)	5 (3%)	5 (2%)
Other ^d^	26 (29%)	68 (47%)	94 (40%)
**Approaches to engaging partners** ^a^ (n, %)			
Patient/stakeholder research team members	74 (81%)	118 (82%)	192 (82%)
➢ Team members as co-investigators ^e^	44 (59%)	63 (53%)	107 (56%)
Advisory groups	72 (79%)	123 (85%)	195 (83%)
Opinion polls, surveys or interviews	39 (43%)	53 (37%)	92 (39%)
Other ^f^	4 (4%)	13 (9%)	17 (7%)
**Study phases in which partners were engaged** ^a^ (n, %)			
Research topics and/or research questions	54 (59%)	90 (63%)	144 (61%)
Interventions and/or comparators	62 (68%)	101 (70%)	163 (69%)
Outcomes and/or measurement	71 (78%)	106 (74%)	177 (75%)
Other aspects of study design	61 (67%)	94 (65%)	155 (66%)
Recruitment and/or retention	53 (58%)	97 (67%)	150 (64%)
Data collection	29 (32%)	64 (44%)	93 (40%)
Data analysis and/or results review	34 (37%)	98 (68%)	132 (56%)
Dissemination	24 (26%)	77 (53%)	101 (43%)

^a^Not mutually exclusive

^b^Defined as a person who is an authority in a particular area or topic

^c^Defined as those who deliver health professional education include public and private universities and colleges, individuals affiliated with the delivery or administration of health professional education, and trade or professional associations representing these institutions, organizations, and individuals (e.g., dean of a nursing school, director of a residency program, and manager of a provider of continuing medical education)

^d^Verbatim descriptions of partners include: biostatisticians, case managers, clinical investigators, community health worker organizations, community-based organizations, community residents, dietitians, educational institutions, National Institutes of Health, nurses, professional organizations/societies, regulatory/compliance professionals, support group organizations, and technology advisors

^e^Asked only to those reporting partners as research team members

^f^Verbatim responses: working with partners on producing and delivering conference presentations, engaging partners in conversations to inform study, partners serving as peer buddies, enlisting partners as pilot study participants, and investigators and partners co-presenting webinars

#### Partner sample

The partner sample included 260 people and was mostly female (70%), white (78%), with an average age of 54 (± 13), and most commonly represented patients/consumers (29%), clinicians (13%), and caregivers (12%), as well as smaller proportions of other stakeholders that PCORI seeks to engage, including advocacy organizations representing patients and/or caregivers and public and private healthcare insurers (Table 3). The year 2 sample included similar absolute numbers of patients/consumers compared to the year 1 sample, along with slightly larger numbers of other types of stakeholders. More than half of partners reported having a university or post-graduate degree. The partner sample represented 124 distinct PCORI research projects. Forty-nine percent of projects in the investigator sample have one or more partners in the partner sample, with one to seven partners reporting per project (mean 2.1 ± 1.3).

**Table 3:** Partner-reported characteristics (N = 260 partner-completed WE-ENACT, representing 124 projects)

Characteristic	Year 1 WE-ENACT (*n* = 123)	Year 2 WE-ENACT (*n* = 137)	Total (*N* = 260)
**Age** (mean ± SD years)	55 (± 13)	54 (± 13)	54 (± 13)
Missing	8	9	17
**Gender** (n, %)			
Female	79 (68%)	96 (73%)	175 (70%)
Male	37 (32%)	36 (27%)	73 (29%)
Transgender	1 (< 1%)	0 (0%)	1 (< 1%)
Missing	6	5	11
**Race** (n, %)			
American Indian/ Alaska Native	0 (0%)	3 (2%)	3 (1%)
Asian	4 (3%)	5 (4%)	9 (4%)
Black or African American	12 (10%)	20 (15%)	32 (13%)
Native Hawaiian or other Pacific Islander	1 (< 1%)	1 (< 1%)	2 (< 1%)
White	95 (80%)	98 (75%)	193 (78%)
Other	7 (6%)	3 (2%)	10 (4%)
Missing	4	7	11
**Ethnicity** (n, %)			
Hispanic/Latino	7 (6%)	5 (4%)	12 (5%)
Missing	5	6	11
**Primary partner community represented** (n, %)			
Patient/consumer	35 (32%)	37 (28%)	72 (29%)
Clinician	18 (16%)	14 (11%)	32 (13%)
Caregiver/family member of patient	12 (11%)	18 (14%)	30 (12%)
Patient/caregiver advocacy organization	17 (16%)	7 (5%)	24 (10%)
Community-based organization	5 (5%)	12 (9%)	17 (7%)
Subject matter expert ^a^	7 (6%)	8 (6%)	15 (6%)
Clinic/hospital/ health system representative	5 (5%)	7 (5%)	12 (5%)
Payer (public or private insurance)	0 (0%)	4 (3%)	4 (2%)
Policy maker (government official)	0 (0%)	2 (2%)	2 (< 1%)
Other ^b^	11 (10%)	23 (17%)	34 (14%)
Missing	14	5	18
**Educational attainment** (n, %)			
Less than high school (did not complete some or all of lower and upper secondary education)	0 (0%)	1 (< 1%)	1 (< 1%)
High school graduate (equivalent to completion of upper secondary education) or General Education Development Completion (alternative completion of high school)	2 (2%)	3 (2%)	5 (2%)
Post high school training other than university (vocational or technical)	3 (3%)	4 (3%)	7 (3%)
Some university attendance	16 (13%)	25 (19%)	41 (16%)
University graduate	28 (23%)	31 (23%)	59 (23%)
Postgraduate education	71 (59%)	69 (52%)	140 (55%)
Missing	3	4	7
**Previously partnered on other research project** ^c^ (n, % yes)	64 (54%) (*n* = 119)	n/a	n/a
**Previously partnered with current investigators** ^c^ (n, % yes)	46 (42%) (*n* = 109)	n/a	n/a
**Time worked in years with current investigators** ^c,c,d^ (mean ± SD)	4.3 ± 3.0) (*n* = 45)	n/a	n/a
**Study phase(s) in which engaged**			
Researcher understanding of patient and stakeholder needs	96 (86%)	102 (77%)	198 (81%)
Research topics and/or research questions	43 (38%)	37 (28%)	80 (33%)
Interventions and/or comparators	44 (39%)	34 (26%)	77 (32%)
Outcomes and/or measurement	62 (55%)	56 (42%)	118 (48%)
Recruitment: Training research staff on how to recruit and work with patients	35 (31%)	23 (17%)	58 (24%)
Recruitment and retention: Finding and/or retaining participants	49 (44%)	43 (33%)	92 (38%)
Data collection	23 (21%)	20 (15%)	43 (17%)
Data analysis and/or results review	39 (35%)	56 (42%)	95 (39%)
Data application to real world settings	34 (30%)	42 (32%)	76 (31%)
Dissemination	22 (20%)	40 (30%)	62 (25%)
Missing	11	5	16

^a^Defined as a person who is an authority in a particular area or topic

^b^Verbatim responses: Advisory panel member; Community-based organization and free clinic/pharmacy; Chair, parent advisory board; Clinical informaticist; Clinical researcher; Clinical social worker; Community advisor; Community partner intermediary and cultural broker; Disparity expert; Executive director of patient foundation; Long term and post-acute care provider trade association; Parent; Parent and leader of advocacy organization; Patient advisor × 2; Patient advisor/co-author; Patient advocate × 2; Patient and caregiver; Patient and research advocate; Patient and subject matter expert; Patient/consumer/caregiver/family member of patient; Patient family and child advocate; Peer group facilitator; Practice based co-PI; Previously a patient; Professional society representative; Project consultant × 2; Research assistant with lived experience; Research expert × 2; Survivor of child abuse

^c^Item only asked at Year 1 WE-ENACT

^d^Item only asked of partner respondents who indicated they previously partnered with the current investigator

### Qualitative findings

#### Overview of findings

We found that engagement challenges are most often described in three domains: (1) infrastructure to support engagement, (2) building relationships, and (3) maintaining relationships. Infrastructure challenges related to the financial and human resources needed to engage research partners, including funding support and dedicated staff time, identifying diverse groups of partners, and juggling real-world logistical demands of partners who may be ill or caring for others. While there are similarities between the two domains of building relationships and maintaining relationships, we present them separately here to highlight the issues faced when initially forming partnerships versus challenges with fostering and nurturing established relationships. Effective communication is a key aspect underlying both relationship domains. Challenges to both building and maintaining relationships relate to having authentic, positive interactions that facilitate mutual understanding, full participation, and genuine influence on the projects. Relationship challenges were particularly salient among projects engaging partners representing hard-to-reach populations and partners who did not speak English proficiently. Most infrastructure challenges persisted across time, with relationship building challenges more prominent in early reports and relationship maintenance challenges more prominent in later reports.

Strategies to prevent or mitigate engagement challenges also corresponded overall to the same three domains. Investigators and partners typically described strategies to facilitate engagement generally rather than specific actions to address particular challenges. While at times, investigators framed effective strategies as time-consuming, they did not report on strategies that they found ineffective or that they abandoned due to time constraints.

We provide detailed information on both challenges and strategies by domain. Tables 4, 5 and 6 summarize the challenge and strategy themes and provide examples of specific strategies for each domain (infrastructure, relationship building, and relationship maintenance), organized by the type of respondent that identified them (investigators, partners, or both). Infrastructure challenges and strategies themes were more of a focus for investigators than partners, while partners described more challenges related to relationship maintenance. Moreover, the challenges related to relationship maintenance differed modestly by stakeholder community (see Table 6).

**Table 4:** Infrastructure challenges and strategies reported by investigators and research partners

Infrastructure			
	General Challenge	General Strategy	Examples of Specific Strategies
**Investigators**	• Needing substantial time and effort to support partners and manage engagement • Identifying partners with diverse backgrounds and perspectives	• Dedicate staff to manage engagement • Integrate partner input for scheduling • Attend to the availability and accessibility of meetings • Appropriately compensate partners	• Account for staff time needed to support coordination, communication, and capacity building needs • Adjust meeting dates and intervals to account for partner work schedules and geographic distance from meeting place • Address partner transportation needs • Employ a range of meeting and communication strategies (email, telephone, conference call, or video call) • Accommodate partners’ lack of internet access or other resources • Be aware that electronic document sharing platforms may not work for all partners • Compensate for partner document review and meeting time, travel time, and transportation costs • Help partners navigate institutional systems (e.g., hiring policies, documentation for compensation)
**Investigators and partners**	• Scheduling and logistics related to partners’ competing demands • Maintaining consistent partner participation	*None described*	*None described*
**Partners**	*None described*	*None described*	*None described*

**Table 5:** Relationship building challenges and strategies reported by investigators and research partners

Relationship building			
	General Challenge	General Strategy	Examples of Specific Strategies
**Investigators**	• Establishing positive relationships with affected communities • Extending authentic invitations to potential partners with diverse perspectives	• Strengthen relationships with affected communities • Ensure participation of partners with diverse perspectives	• Develop relationships with advocacy organizations to build community trust and to identify and invite partners • Increase community awareness about research areas and funded studies by posting on community-oriented blogs, discussion boards, and social media sites • Integrate breakout sessions by language group • Set meeting agendas where the historically least represented or most marginalized groups speak first and last
**Investigators and partners**	• Ensuring partner and research team cohesion	• Engage partners early and consistently • Orient, train, and build capacity	• Develop project roadmaps to describe phase of the project and where the team and work are headed • Hold pre-meetings with patient and caregiver partners to prepare • Create mentoring opportunities for partners
**Partners**	*None described*	• Connect partners to the research team	• Have face-to-face social gatherings in addition to project-oriented meetings • Create resource with all team and partners’ photos and biographies

**Table 6:** Relationship maintenance challenges and strategies reported by investigators and research partners

Relationship maintenance			
	General Challenge	General Strategy	Examples of Specific Strategies
**Investigators**	• Being responsive to diverse partner perspectives and using their guidance	• Develop group facilitation skills • Adapt engagement goals in response to partners’ needs	• Practice or learn skills in active listening, giving everyone a voice, facilitating respectful dialogue, navigating differences of opinion • Ensure research project goals match the capabilities and priority populations served by partner organizations
**Investigators and partners**	• Having shared language of research terminology and concepts • Maintaining diverse partner representation	• Use accessible language • Communicate frequently • Consistently communicate value of partners’ contributions	• Create a glossary of research and health terms • Send meeting notes regardless of meeting attendance • Ensure regular communication to partners to describe partners’ influence on study and value to the research process (e.g., newsletter, standing agenda item)
**Partners**	• Managing expectations about project progress • Experiencing perspective as understood and valued^a^ • Knowing impact of contribution^a^	• Clarify roles and expectations throughout the project	• Clearly define the purpose of partnerships and partner roles • Create opportunities to define new partner roles and recalibrate expectations across study course

^a^ Challenge theme was more prominent among respondents who identified as patients/consumers, caregivers/family members, representative of patient/caregiver advocacy organizations, and representatives of community-based organizations compared to other stakeholder respondents (i.e., clinicians, representative of clinics, hospitals, health systems, payers, policymakers, and subject matter experts)

#### Infrastructure challenges

##### Needing substantial time and effort to support partners and manage engagement

Investigators reported needing substantial time and effort for tasks including scheduling meetings; addressing partner transportation needs; helping partners navigate institutional systems; communicating with and building partners’ research literacy and capacity; incorporating partner feedback; and engaging partners in shared decision-making.

##### Identifying partners with diverse backgrounds and perspectives

Investigators also reported challenges in identifying partners due to underdeveloped connections to underserved groups (e.g., racial and ethnic minorities, lower-income, less educated) as well as to young men and primary care providers. Moreover, some investigators perceived hesitancy among potential partners due to limited knowledge about research or alternative prevention or treatment approaches—for example, testing a behavioral intervention to manage chronic pain compared to a commonly used pharmaceutical approach.

##### Scheduling and logistics related to partners’ competing demands

Both investigators and partners described challenges with scheduling and logistics that led to adjusting meetings and events to different dates and intervals. Partners also described how meetings were sometimes scheduled at inconvenient locations and times, such as during work hours. This challenge extended to finding time for large in-person convening to discuss issues thoroughly and accounting for travel time for geographically distant partners. Capturing the sentiment of many, one investigator said, “This is not a challenge but a reality of participatory research.”

##### Maintaining consistent partner participation

Both groups noted challenges maintaining consistent participation was difficult at both the individual and organization levels (e.g., when a partner represented a patient advocacy group or community-based organization). Partners often faced unpredictable demands stemming from their own health conditions or caregiving, complicating logistics and their research participation. For example, a patient/consumer partner explained “For the last several months … I have had health problems and my response time in providing requested input has been sluggish at times.” Other factors hindering consistent participation included some partners’ lack of internet access or other resources, administrative delays such as for finding a document sharing platform that worked well for all partners, and mismatches between project goals and capabilities of partner organizations (e.g., a partner organization offered to help identify and enlist partners but did not have access to the relevant population; community organizations’ preexisting commitments to other projects interfering with taking on new partnerships).

#### Strategies to prevent and mitigate infrastructure challenges

##### Dedicate staff to manage engagement

Investigators described dedicating staff to manage engagement, including restructuring project staffing to build partner research literacy and capacity; share study information with partners (e.g., via email, newsletters, or a project website); offer additional pre- and post-meetings to discuss study progress; integrate partner guidance into study design and conduct; and ensure positive relationships with affected communities. One investigator shared how multiple research team members contacting partners caused confusion, saying, “We now anticipate when this overlap may occur and select one coordinator to serve as the main point of contact.” While this strategy required more staff cross-training, the approach offered more consistency to partners.

##### Integrate partner input for scheduling

Investigators described incorporating partner input to agree on meeting time, location, frequency, and ways to meet (e.g., by email, webinar, individual consultation).

##### Attend to the availability and accessibility of meetings

Relatedly, investigators also described enhancing other aspects of meeting accessibility by responding to partner preferences; using individual or small heterogeneous group meetings; and sending meeting notes regardless of attendance.

##### Appropriately compensate partners

Investigators described compensating partners with monetary and other incentives for partners’ effort and transportation costs.

#### Relationship building challenges

##### Establishing positive relationships with affected communities

Investigators described the challenge of establishing positive relationships with affected communities when community members and research staff are unfamiliar with one another. For example, they described that some communities expressed fatigue or skepticism based on previous research experiences where they had little input and/or experienced little benefit. Moreover, some investigators described how project community outreach efforts were slowed or caused confusion when there is overlap between roles or responsibilities as a research partner (e.g., recruit study participants into diabetes intervention) and their professional obligations at an advocacy or community-based organization (e.g., health promotion and disease prevention related to diabetes).

##### Extending authentic invitations to potential partners with diverse perspectives

Another investigator-cited challenge was conveying respectful and genuine invitations to engage on research projects that would not be perceived as tokenistic by affected communities that might feel over-studied and/or under-served.

##### Ensuring partner and research team cohesion

Both investigators and partners cited concerns about team cohesion. For example, a clinician partner described the need to “collaborate more effectively—such as doing more team-building meetings, putting in time to consult with each other about tough situations.”

#### Strategies to prevent and mitigate relationship-building challenges

##### Strengthen relationships with affected communities

Both investigators and partners described strengthening relationships with affected communities, including individuals and organizations. Many study teams developed and leveraged relationships with advocacy organizations to build overall trust, identify and invite partners, inform the community about the research project, and network with other researchers and experts in the field. Some investigators described how partners represented the project at professional or community events. Several teams also used the internet to develop awareness among affected communities more broadly by posting information about the project on blogs, discussion boards, and social media sites.

##### Connect partners to the research team

Partners described strategies to more strongly connect partners to the research team, including face-to-face social and project-oriented gatherings and directories with photos and biographies. For example, a patient/consumer partner noted that “a booklet with photos and biographies of the team members would probably help the other patients and caregivers. Unlike the medical professionals on the team, most of the patients and caregivers are home-bound.”

##### Orient, train, and build capacity

Both investigators and partners described empowering partners and creating a culture of open dialog through orienting, training, and offering ongoing capacity-building opportunities. As one investigator described, “During the first six months, we met with patient stakeholders prior to each conference call to make sure they felt comfortable with all of the information and had a strong voice. We no longer need to do this as they are truly part of our stakeholder advisory committee.” A partner representing an advocacy organization recommended “real-time mentoring—find new advocates to serve on the project with trusted experienced advocates so that they can see in real time what it looks like.”

##### Ensure participation of partners with diverse perspectives

Investigators described ensuring participation of partners with diverse perspectives by adapting meeting formats to encourage participation among an array of partners within group settings, such as youth and partners with primary languages other than English. Some teams used breakout sessions by language group, while others altered meeting agendas to allow partners representing more vulnerable groups to speak first.

##### Engage partners early and consistently

Both investigators and partners described engaging partners early and consistently, including regularly involving partners in multiple stages of the research process. One investigator stated, “We have learned the lesson that we need to communicate earlier and more often to help research team members internalize the key points of the study. We have developed project ‘roadmaps’ to continually cover where we are in the project and where we are going.” Partners explained that regular meetings helped them understand project progress and provide more relevant guidance.

#### Relationship maintenance challenges

##### Having shared language of research terminology and concepts

Both groups described lacking a shared language. For example, a patient/consumer partner stated, “I don’t want to disrupt the momentum of the conversation for a definition.” Similarly, a clinician partner said, “Coming from [different] disciplines, we speak a different ‘language’ and see things very differently, which is why more team building and communication training would have been helpful.”

##### Managing expectations about project progress

Relatedly, partners described frustrations about slower than expected project progress, including lengthy IRB approval, loss of momentum because of staff turnover, and slow participant enrollment.

##### Maintaining diverse partner representation

Both groups described difficulty in maintaining diverse partner representation, especially partners from communities historically underrepresented in the partnering in research (e.g., adolescents, male patients, male caregivers, partners representing racial and ethnic minority groups, lower income groups). Partners described how a diversity of perspectives was rooted in enlisting qualified and committed partners. While this challenge intersects with maintaining consistent partner participation, it underscores the social and historical context of underrepresented groups and why the engagement process and project goals may not resonate over time and, in turn, lead to waning partner motivation to engage.

##### Being responsive to diverse partner perspectives and using their guidance

Investigators described the challenge of being responsive to diverse partner perspectives, further exacerbated by disparities in education, collaboration skills, and research literacy, that resulted in conflicts, delays, or uncomfortable meeting dynamics. Investigators indicated these tensions stem from partner suggestions outside of the scope of the research aims, the sharing of partners’ personal experiences that did not appear to be directly connected to the health topic, and other suggestions that investigators felt were otherwise difficult to integrate into the planning and conduct of the study. As one investigator shared, “We have found patient [partner] feedback is constant, even after study activities have begun. However, incorporating new feedback is often difficult once formal data collection activities have begun.” Other challenges included managing conflicting feedback from different partners (e.g., different partners view the same study participant materials as too long, too short, and just right). Extending this theme, investigators were concerned about how to respectfully inform partners that they could not act on all suggestions.

##### Partners experiencing their perspectives as understood and valued

Similarly, partners, particularly patients and caregivers, described not always experiencing their perspectives as understood and valued, and they indicated this was a key factor in decisions to stay involved.

##### Partners knowing the impact of their contribution

Relatedly, partners, particularly patients and caregivers, also described not knowing the impact of their contribution to the research project*.* One patient/consumer partner described the absence of a communication feedback loop, saying, “Sometimes, I am not sure that my contribution is helpful or hitting the ‘target’ for the researchers.”

#### Strategies to prevent and mitigate relationship-maintenance challenges

##### Clarify roles and expectations throughout the project

Partners described clarifying roles and expectations. A partner representing an advocacy organization explained, “Don’t have a research partner in the room just for the sake of saying that the team had a patient present. Define the purpose, the role, the input you’re hoping to receive.” Partners described this strategy as important both at the beginning and as the project progresses and partner skills evolve.

##### Adapt engagement goals in response to partners’ needs

Investigators described adapting engagement goals in response to partners’ needs, often resulting in modifications to project plans and timelines. One investigator shared, “We had planned on the hospice volunteer stakeholders doing community outreach to oncology offices, churches, and community organizations, but the hospices indicated that their marketing plan would not accommodate such outreach … They have asked us not to engage in the planned activities and we respectfully adhere to the request of our hospice partners.”

##### Use accessible language

Both groups, in very practical terms, described using accessible language and avoiding research jargon when inviting partners to engage and throughout the research activities. Investigators reported that these communication strategies streamlined input and increased collaboration, and partners said such efforts created a more inclusive environment.

##### Develop group facilitation skills

Relatedly, investigators described developing group facilitation skills to create more harmonious and productive group dynamics and encourage more diverse partner perspectives.

##### Communicate frequently

Moreover, both groups also described communicating frequently as an important resource-intensive strategy to keep partners engaged and to ensure actionable guidance. This strategy included frequent meetings and communication with partners, ranging from weekly to biweekly to monthly to quarterly as needed. As one patient/consumer partner pointed out, getting information with enough notice allows time “to discuss it and make real change.”

##### Consistently communicate the value of partners’ contributions

Investigators and partners both described consistently communicating the value of partners’ contributions to the project as a key aspect of engagement, with one partner saying, “Providing detailed responses to feedback so that we know our work is valued is the most important part.” A caregiver/family member partner described how “roundtable discussions where every person’s ideas were valued equally were really important. It made it so that everyone was more willing to share honestly because they weren’t intimidated or made to feel that the doctors/researchers knew better than we as parents/caregivers did.” An investigator echoed this sentiment, describing how the project sends a quarterly update to stakeholders about the study’s progress and how partner suggestions contributed to the project. A subset of investigators described using visual tools, such as roadmaps, to address multiple needs including orienting partners to the project, facilitating capacity-building opportunities throughout the project, and consistently communicating with partners about how their contributions shaped the project.

## Discussion

This study offers unique insights about challenges and strategies for engagement that help fill important information gaps. To our knowledge, this study is the largest to date to explore real-world engagement challenges and strategies, and in particular, is first to include a large group of engaged partners. The partner perspective helps the field focus on the challenges and strategies that are most important to patient and other stakeholder partners, which are critical for effectively enhancing engagement and cannot be learned from studying investigators alone. This study also provides more depth and detail about engagement challenges and how they can be addressed in research settings. Finally, PCORI represents the largest effort in the US to support engagement, and learning from PCORI-funded research teams amplifies and expands themes that are common across different contexts and countries. In addition to being an understudied context, given the inclusion of other healthcare stakeholders alongside patients, caregivers, and the public in PCORI-funded projects, our study offers an opportunity to understand how investigators balance the needs of different stakeholders while preserving the patient voice.

At a high level, our finding that challenges and strategies related to adequate infrastructure, including logistical support and resources, as well as building and maintaining relationships, supports the prevailing recognition that resources are necessary but not sufficient for effective engagement. Both researchers and partners underscored the relationship-based nature of engagement and principles thought to underly engagement such as reciprocity, co-learning, trust, and transparency [34].

While the investigators and partners shared many themes on challenges and strategies, each group offered unique perspectives. The relationship maintenance themes would not have been as prominent without the partner perspectives. Patients, caregivers, and stakeholders from the healthcare field encountered many of the same challenges despite differences in experience and types of expertise. Partners offered unique views related to how their competing life demands, including managing their own health condition or caring for others, often prevent consistent participation or timely feedback despite a desire to be involved, calibrating expectations related to the relative slow-going nature of research, and gaining clarity about their roles and contributions to the projects. Investigators voiced challenges and strategies to identify and extend genuine invitations to partners with diverse backgrounds and perspectives, to appropriately compensate partners, and to manage conflicting partner feedback. While the latter challenge is salient to group work in general, it may be amplified as individuals with a wider range of backgrounds are included in research teams. For many investigators, it seemed that such competencies went beyond their daily research work and required relationship management and collaboration that they are not yet accustomed to, have the skills for, or receive adequate support.

Compared to past studies, we found greater focus on incorporating partner input on scheduling, accessibility of meetings, and managing expectations about project progress. We also found emphasis on building team cohesion, fully integrating partner feedback, and being responsive to diverse partner perspectives. Many other themes also amplify past observations based on individual projects or small samples of projects (e.g. [13, 15, 29–33],) suggesting these challenges and strategies reflect key issues for engagement in a variety of settings and contexts, including US healthcare systems. Overall, our findings provide tangible ways for research teams to practice four of the six UK Standards for Public Involvement [44]: inclusive opportunities, working together, support and learning, and communications. For example, the strategies of improving group facilitation skills, using plain language, and consistently communicating the value of partners’ contributions reflect the Communications standard (see supplemental Appendix B for a full mapping of the general strategies observed in this study to the UK Standards for Public Involvement). The fact that infrastructure and relationship challenges were so prominent in the context of PCORI – a funder with an emphasis on stakeholder partner engagement - highlights the continuing need to support engagement with greater resources and guidance.

### Implications

Our findings point to implications for research policy and practice. Comprehensively addressing engagement challenges requires contributions from multiple levels, including institutions and funders. To mitigate infrastructural challenges, funders and institutions need to institute policies and resources (e.g., appropriate proportion of study budget for managing engagement activities and compensating partners, hiring policies at academic institutions to make it easier for research teams to employ patients or caregivers as staff on the research team), create incentives for engagement (e.g., tenure policies that value fostering partnerships), and allow more flexibility and time to be responsive to stakeholder-informed changes to research plans (e.g., contracts with funders, ethical review board). There is an inherent tension between the time associated with this flexibility and the pressure to generate and share timely results that can inform clinical practice. Such policies and guidance should also foster diversity and inclusivity of a broad spectrum of the public by working with individuals and organizations not historically invited to prioritize research questions and co-create scientific evidence.

It is also critical to develop and test new models to make engagement more efficient and accessible – especially for partners who have been historically underrepresented in the research enterprise. Such models could go beyond commonly utilized one-person, one-vote methods (e.g., research team members, advisory board approaches) and include practical evaluations of both engagement processes and outcomes so that real-time adjustments can be made. Further, training tools and capacity building are needed for content knowledge and honing researchers’ and partners’ relationship and communication skills. These skills include group facilitation, deliberative processes, and shared terminology.

PCORI’s more recent resources and guidance on engagement are directly responsive to the findings we present here. These resources and guidance are also in line with PCORI’s priority to eliminate disparities in health and healthcare outcomes, in part, by engaging an ever-more diverse range of stakeholders [45]. PCORI has developed repositories to increase access to engagement related tools and literature (Engagement Tool and Resource Repository for Patient-Centered Outcomes Research [46], Engagement in Health Research Literature Explorer [47]. PCORI has or will soon launch training modules on research fundamentals [48], how to work in teams, and empowering researchers and partners to more effectively collaborate on data planning, analysis, and interpretation. Additionally, PCORI now requires investigators to submit an updated engagement plan [49], early after project start, that includes many components that map to the strategies described in this study (e.g., engagement roles and expectations, orientation and capacity building, communication feedback loops).

Our findings also have implications for researchers and partners practicing engagement, beyond the specific strategies offered by these investigators and partners. Awareness of commonly encountered challenges enables expectation-setting and honest communication about the demands of partnership. The strategies presented here to facilitate the integration of underrepresented groups’ values, interests, and preferences in health research are applicable to both groups and individuals already engaged and those yet to partner in research. The findings also highlight the importance of flexibility and willingness to try creative solutions to facilitate engagement.

Future research should seek to better understand the challenges experienced by partners with different educational levels, health literacy, research use, and other socio-demographics and how these factors contribute to engagement experiences. Such findings could improve greater inclusion and diversity of stakeholder partners by unearthing the ways that current strategies to identify, enlist, and involve partners may perpetuate or exacerbate underrepresentation of underserved communities. Additional inquiry is also needed on how different types of stakeholders, including those who work in the healthcare field, may change the power dynamics in conversations and more generally within a research team. Future research should also understand challenges and strategies that emerge in later phases of research, as well as strategies attempted that were deemed unsuccessful or impractical, how challenges and strategies evolve over the course of projects and study team relationships, and how they compare to those described here for the earlier phases. Further, a study comparing perspectives on engagement challenges and strategies between individual investigators and partners working together would help to reveal further opportunities for improved communication and collaboration in project teams. Ultimately, to facilitate optimal engagement, the field must identify which strategies work best for whom and under what circumstances, including how these strategies affect research team relationships, satisfaction with the engagement process, and the quality and relevance of the resulting research.

### Limitations

Consistency between engagement challenges and strategies found in this study and those described by earlier work, including the UK Standards for Public Involvement, encourages confidence in these findings. Nonetheless, there are some important limitations. First, data were self-reported, which could bias responses toward more memorable or recent experiences and recall may have been limited. Although social desirability may limit accuracy and/or comprehensiveness of responses provided to the research funder, we did obtain a range of positive and negative responses, and nearly all respondents provided information about engagement challenges.

There were practical challenges associated with including partner perspectives. We do not know investigators’ reasons for choosing whether to nominate partners to complete the WE-ENACT survey, why they chose to invite specific partners, nor why some partners did not complete the WE-ENACT. The investigators may have nominated partners they believed were more likely to complete the WE-ENACT or respond favorably. Responding partners, first nominated by the investigator, had to have enough time, energy, interest, trust in the research team or funder, and comfort with the English language to complete the WE-ENACT. Given our sampling and data collection approaches, results may not reflect perspectives of all types of research partners. We had limited diversity in terms of race, ethnicity, education, sex, and gender (i.e., a majority of responding partners were white more formally educated women).

Moreover, these respondents (which reflect nearly all PCORI funded CER projects at the time of the analysis and 37% of CER projects funded to-date) are from PCORI’s earliest funded CER projects, many of which were funded before PCORI offered more engagement resources and before PCORI’s shift toward larger, more targeted projects. More recent projects may have had different experiences with engagement. Nonetheless, this paper offers important insights, particularly by learning directly from a large sample of partners, that both provide new perspectives and information and validate past research.

## Conclusions

Meaningful engagement of patients and other stakeholders throughout the research process comes with challenges, as does any innovation in high impact research processes like rigorous methods, ethical review, and safety monitoring. Evidence suggests, though, that engagement also helps to overcome other challenges that historically lead to research waste [50] by influencing research relevance to healthcare decisions, acceptability to patients and clinicians, and feasibility to complete trials (e.g. [9, 12, 15, 29]). Despite the potential for engagement of patients and other stakeholders to improve research, engagement remains relatively uncommon in clinical research [16, 17]. A more robust evidence base about engagement methods and impact could facilitate research funders and institutions, researchers, and community members in viewing engagement as an investment in creating and translating more useful research to ultimately improve patient care and outcomes. PCORI funding enabled engagement of patients and other stakeholders on a scale that has not been done before in the US. Our study of these investigators’ and partners’ experiences offers an opportunity to inform efforts to more effectively address the challenges related to engagement infrastructure, relationships, and communication, and to more deeply embed patients and other stakeholders in the research process. Further progress towards making patient and other stakeholder engagement in research more widely practiced can be made if researchers, stakeholders, institutions, funders, and policy makers learn together about the key challenges and act together through further research, capacity building, and policy change.

## Supplementary information

**Additional file 1.** Supplemental Appendix A. Questions Used for the Current Analysis. Supplemental Appendix B. Comparison of UK Standards of Public Involvement to the PCORI Findings on Engagement Strategies.

## Data Availability

The datasets analyzed during the current study are not publicly available because they are confidential progress reports and research partner surveys submitted to and managed by the funder.

## Abbreviations

PCORI: Patient-Centered Outcomes Research Institute
US: United States
CER: Comparative effectiveness research
IRB: Institutional review board
WE-ENACT: Ways of Engaging-ENgagement ACtivity Tool
PEAP: PCORI’s Advisory Panel on Patient Engagement
