# Supplementary material for: Researchers, patients, and other stakeholders’ perspectives on challenges to and strategies for engagement
Source: Res Involv Engagem. 2020 Oct 7;6:60. doi: 10.1186/s40900-020-00227-0 (PMC7539495; doi:10.1186/s40900-020-00227-0)
Supplement: Supplementary file 1 — Additional file 1. Supplemental Appendix A. Questions Used for the Current Analysis. Supplemental Appendix B. Comparison of UK Standards of Public Involvement to the PCORI Findings on Engagement Strategies. [file 40900_2020_227_MOESM1_ESM.docx]

**Supplemental Appendix A**

**Questions Used for the Current Analysis**

**Annual Progress Report: question used for investigator-reported data on engagement challenges and strategies**

- Please feel free to share any other information about engagement in your project – challenges, strategies you’ve identified to facilitate work with research partners, positive and negative impacts on your work.

**Mid-year Progress Report: questions used for investigator-reported data on engagement challenges and strategies**

- Challenges with project progress including anticipated upcoming challenges (e.g., delays in IRB approval, delays in recruitment of sites, participant retention issues).
  - How have you overcome these challenges?
  - What is your continued plan for overcoming these challenges?
- Describe progress on your approved engagement plan during the current 6-month reporting period, relative to the activities outlined in your application. Refer to Methodology Standard PC-1 and describe how this standard is being met.
- Describe challenges with patient and stakeholder engagement?
  - How have you overcome these challenges?
  - What is your continued plan for addressing these challenges?

Ways of Engaging-ENgagement ACtivity Tool (WE-ENACT): questions used for partner-reported data on engagement challenges and strategies

- Sometimes there are challenges when researchers, patients, and other stakeholders work together. These might include finding a convenient time to meet or communicating clearly with each other. What have been the biggest challenges for you on this research project?
- What aspects of working on this project did you not like?
- Please share anything else that helped you contribute to this research project in the last 12 months. For example, this may include things the researchers did to ensure everyone was included or things you did to ensure your view was heard.
- Based on your experience with this research project, what would you suggest to be done to help others contribute as research partners?

Note: Other open-ended questions contained in the tools above include engagement activities and partner influence by study phase. Relevant results are published elsewhere (11). Some items underwent modest wording changes for clarification during the study time period.

**Supplemental Appendix B**

**Comparison of UK Standards of Public Involvement to the PCORI Findings on Engagement Strategies**

| **UK Standards**  (November 2019) | **UK Standards**  **Reflection Questions** | **PCORI Findings on**  **Engagement Strategies** |
| --- | --- | --- |
| INCLUSIVE OPPORTUNITIES  Offer public involvement opportunities that are accessible and that reach people and groups according to research needs.  Research to be informed by a diversity of public experience and insight, so that it leads to treatments and services which reflect these needs. | Are people affected by and interested in the research involved from the earliest stages?  Have barriers to involvement, such as payment for time or accessible locations for meetings been identified and addressed?  How is information about opportunities shared, and does it appeal to different communities?  Are there fair and transparent processes for involving the public in research, and do they reflect equality and diversity duties?   - Is there choice and flexibility in opportunities offered to the public? | - Infrastructure: Dedicate staff to manage engagement [investigators] - Infrastructure: Integrate partner input for scheduling [investigators] - Infrastructure: Attend to the availability and accessibility of meetings [investigators] - Infrastructure: Appropriately compensate partners [investigators] - Relationship building: Strengthen relationships with affected communities [investigators] - Relationship building: Ensure participation of partners with diverse perspectives [investigators] |
| WORKING TOGETHER  Work together in a way that values all contributions, and that builds and sustains mutually respectful and productive relationships.  Public involvement in research is better when people work together towards a common purpose, and different perspectives are respected. | Has the purpose of public involvement been jointly defined and recorded?  Have the practical requirements and arrangements for working together been addressed?  Have all the potential different ways of working together been explored, and have these plans and activities been developed together?  Is there is a shared understanding of roles, responsibilities and expectations of public involvement?  Have individuals’ influence, ideas and contributions’ been recognised and addressed? | - Relationship building: Engage partners early and consistently [investigators & partners] - Relationship building: Connect partners to the research team [partners] - Relationship maintenance: Clarify roles and expectations throughout the project [partners] |
| SUPPORT AND LEARNING  Offer and promote support and learning opportunities that build confidence and skills for public involvement in research.  Remove practical and social barriers that stop members of the public and research professionals from making the most of public involvement in research. | Is there a range of support to address identified needs?  Have specific resources been designated to support learning and development opportunities for both the public, researchers, and staff?  Do the public know where to go for information and support about public involvement?  Is there a culture of learning by doing, building on and sharing that learning for researchers, staff and the public? | - Relationship building: Orient, train, and ongoing capacity-building opportunities [investigators & partners] - Relationship maintenance: Adapt engagement goals in response to partners’ needs [investigators] |
| COMMUNICATIONS  Use plain language for well-timed and relevant communications, as part of involvement plans and activities.  Communicate with a wider audience about public involvement and research, using a broad range of approaches that are accessible and appealing. | Has a communications plan been developed for involvement activities?  Are the needs of different people being met through inclusive and flexible communication methods?  Are processes in place to offer, gather, act on and share feedback with the public?  Are you sharing your public involvement learning and achievements, good and bad? | - Relationship maintenance: Develop group facilitation skills [investigators] - Relationship maintenance: Use accessible language [investigators & partners] - Relationship maintenance: Communicate frequently [investigators & partners] - Relationship maintenance: Consistently communicate value of partners’ contributions [investigators & partners] |
| IMPACT  Seek improvement by identifying and sharing the difference that public involvement makes to research.  Understand the changes, benefits and learning gained from the insights and experiences of patients, carers and the public. | Are the public involved in deciding what the assessment of impact should focus on, and the approach to take?  Is it clear what information to collect to help assess impact, including who has been involved and how?  Are there processes in place to help reflect on public involvement?  Are the changes, benefits and learning resulting from public involvement acted on? | *Not applicable* |
| GOVERNANCE  Involve the public in research management, regulation, leadership and decision making.  Public involvement in research governance can help research be more transparent and gain public trust. | Are public voices heard, valued and respected in decision making?  Are public involvement plans in place that are regularly monitored, reviewed and reported on?  Is there visible and accountable responsibility for public involvement throughout the organisation?  Are realistic resources (including money, staff, time) allocated for public involvement?   - Is the privacy of personal information protected by collecting and using it in a suitable way? | *Not applicable* |
